# Genome-Wide Identification of Candidate Genes Associated with Heat Stress in Mulberry (*Morus alba* L.)

**DOI:** 10.3390/cimb45050264

**Published:** 2023-05-08

**Authors:** Xin Jin, Michael Ackah, Adolf Acheampong, Qiaonan Zhang, Lei Wang, Qiang Lin, Changyu Qiu, Weiguo Zhao

**Affiliations:** 1 Jiangsu Key Laboratory of Sericultural Biology and Biotechnology, School of Biotechnology, Jiangsu University of Science and Technology, Zhenjiang 212100, China; jinxin9502@126.com (X.J.); loer9725@126.com (Q.Z.); wangleiwendy@126.com (L.W.); 2 School of Food and Biological Engineering, Jiangsu University, Zhenjiang 212013, China; 3 Guangxi Sericultural Research Institute, Guangxi Zhuang Autonomous Regin, Nanning 530007, China; gxlq67@163.com (Q.L.); changyuqiu2008@163.com (C.Q.)

**Keywords:** mulberry plants, heat stress, RNA-Seq, transcription factors, differential expressed genes

## Abstract

Mulberry (*Morus alba* L.) is an economically important plant for the silk industry and has the possibility of contributing immensely to Chinese pharmacopeia because of its health benefits. Domesticated silkworms feed only on mulberry leaves, meaning that the worms’ survival depends on the mulberry tree. Mulberry production is threatened by climate change and global warming. However, the regulatory mechanisms of mulberry responses to heat are poorly understood. We performed transcriptome analysis of high-temperature-stressed (42 °C) *M. alba* seedlings using RNA-Seq technologies. A total of 703 differentially expressed genes (DEGs) were discovered from 18,989 unigenes. Among these, 356 were up-regulated, and 347 were down-regulated. KEGG analysis revealed that most DEGs were enriched in valine, leucine and isoleucine degradation, and in starch and sucrose metabolism, alpha-linolenic acid metabolism, carotenoid biosynthesis and galactose metabolism, among others. In addition, TFs such as the NAC, HSF, IAA1, MYB, AP2, GATA, WRKY, HLH and TCP families were actively involved in response to high temperatures. Moreover, we used RT-qPCR to confirm the expression changes of eight genes under heat stress observed in the RNA-Seq analysis. This study provides *M. alba* transcriptome profiles under heat stress and provides theoretical bases to researchers for better understanding mulberry heat response mechanisms and breeding heat-tolerant mulberry plants.

## 1. Introduction

The mulberry (*Morus alba)* plant is a very significant crop, especially for the sericulture industry. The leaves of mulberry are crucial for feeding silkworm insects; therefore, silkworm growth depends on the quantity and quality of the leaves [1]. Mulberry survives in various conditions (50° N and 50° S latitudes) [2]. However, over the years, temperatures (cold and heat) have been a major setback for the sericulture industry because they appear to inhibit the growth of the mulberry tree [2].

Temperature stress causes damaging consequences on cell division, which leads to a detrimental effect on growth and development [3]. Most plants are susceptible to temperature changes during the flowering phase, affecting pollen quality and productivity [4]. Genes, including those (P5CS2: delta-1-pyrroline-5-carboxylate synthase 2, PETC: photosynthetic electron transport chain, HSP90.1: 90-kDa heat shock protein, TOC1: two-component response regulator-like APRR1 and J8: DnaJ-J8) associated with high temperatures (heat stress) have been reported [5]

Mulberry seedlings experience drastic morphological changes when exposed to a prolonged period of high temperature and drought stress [6]. Such morphological changes include thin leaves and leaf color slowly switching from a natural green to light green and then to a yellow color. In addition, heat stress affects metabolic and cellular processes and morphological changes [7]. For instance, heat stress can decrease enzyme activities, trigger protein kinases, and cause the overexpression of some chaperones, such as HSP [7]. Some genes change their expression patterns when exposed to heat stress [8]. This pattern shift is a vital stage in initiating the transfer of mechanisms that can enable the plant to moderate or manage external stress to avert catastrophe [8].

Global temperatures are anticipated to increase substantially by 2 °C or more by the end of the century [9]. Owing to this prediction, a deeper understanding of how plants (mulberry) control external stress is vital for plant sustainability. Research has been conducted on model plants to define the transcriptomic changes of heat stress in recent years, subsequently improving those model plants [5]. *M. alba* is an economically important crop for the sericulture industry, but no research has been conducted to reveal the transcriptomic changes of mulberry under heat or high-temperature stress, making the breeding of heat-tolerant mulberry difficult. Owing to this gap, it becomes necessary to conduct transcriptome analysis to explore genes that respond to heat and identify novel genes that might help improve mulberry tolerance to heat stress.

RNA-Sequencing, over the years, has become an accepted tool to explore the transcriptomes of many organisms, from microalgae to plants and animals [10]. RNA-Seq has been primarily used to identify novel and conserved stress-responsive genes, particularly those related to biotic stress tolerance [11]. Some economical crop species, such as rice [12], maize [13] and potatoes [14], have made extensive use of this technology to expose genes that are responsive to abiotic stresses. Although silkworms’ lives largely depend on mulberry plant leaves for survival [15], no transcriptome work concerning heat-stressed mulberry has been conducted. On this account, RNA-Seq was employed to uncover genes and transcription factors that respond to heat stress in this article.

This article seeks to uncover the gene players that respond to heat stress in *M. alba* and lay the foundation for feature research on functional genomics on putative genes in response to heat stress in *M. alba,* which can be crucial in the breeding of heat-tolerant mulberry.

## 2. Materials and Methods

*M. alba* (Yu-711) used in this study was obtained and raised at the Sericulture Research Institute of the National Mulberry Gene Bank, Chinese Academy of Agricultural Sciences, Zhenjiang, Jiangsu Province, China. The material preparation and raising of the mulberry seedlings followed our previous article [16] with some modifications (supplemented with a growth medium; the seedlings were watered daily and supplemented with an MS culture medium solution containing 4.37 g of MS media dissolved in 1000 mL (pH = 7.0) every three days for 7 days and were then treated with deionized water for 7 days after the leaves were fully grown). When the new shoots reached 20 cm of growth, the plants were then grouped randomly according to our previous study [16]. In total, 18 pots were separated into three groups, with each group consisting of 6 pots. Each group was further subdivided into 2 pots, each containing two seedlings in a growth pot, serving as a biological replicate to observe growth performance. After shoot growth, the pots with the most optimal growth conditions were subjected to both heat stress and control treatment. In order to create the heat stress treatment, one group consisting of 3 pots as a technical replicate and 2 seedlings as a biological replicate was subjected to a temperature of 42 °C in a growth chamber, and the other group was exposed to a normal temperature of 25 °C. Leaf samples were collected daily until the fourth day of heat exposure, when the leaves displayed signs of heat stress. Leaf samples were obtained from both groups and were immediately stored in a –80 °C freezer for further analysis.

### 2.1. RNA-Sequencing and Data Analysis

Total RNA was isolated from two replicates of the heat stress treatment (LH) and two replicates of the control treatment (LRTM) using RNAiso Plus reagent (Takara, Beijing, China) as directed by the manufacturer. RNA degradation and purity were monitored and checked on 1% agarose gels and the NanoPhotometer^®^ spectrophotometer, respectively. Bioanalyzer 2100 systems’ RNA Nano 6000 Assay kit (Agilent Technologies, Califonia, CA, USA) was used to analyze the RNA integrity. The resultant was mixed in RNase-free water to prevent any degradation and then used to build a transcriptome sequence library using the NEBNext Ultra RNA Library Prep Kits for Illumina (NEB, San Diego, CA, USA) following the manufacturer’s instructions. Illumina Hiseq 2500 (Novogene, Beijing, China) was employed to sequence the cDNA libraries. To obtain clean reads, Ploy-N or adapters, as well as low-quality reads, were removed from the raw data. The Q20, Q30 and GC content of the clean data were determined. The clean data were then aligned to the *Morus notabilis* reference genome assembly, ASM41409v2, using HISAT2 v2.0.5 software with the default settings [17]. The clean read numbers mapped to each gene were counted using HTSeq v0.6.1 based on the length of the gene and the number of reads mapped to it. The quantification of gene abundance was achieved by assembling the mapped reads of each sample using StringTie v1.3.1 [18] in a reference-based approach. For each transcription region, an FPKM (fragment per kilobase of transcript per million mapped reads) value was calculated to quantify its expression abundance and variations, using RSEM [19]. Differential expression analysis for two parameters (heat and normal) was undertaken using the edgeR software package v3.2.4 [20]. The false discovery rate was controlled by adjusting the resulting *p*-values using Benjamini and Hochberg’s method. Genes were defined as differentially expressed with the criteria of a fold change of FC > 0 and a false discovery rate of FDR < 0.05. Gene Ontology (GO) analysis was carried out using GOseq software, and the KEGG database was used to analyze the DEG pathways. The statistical enrichment of differential expression genes in the KEGG pathways was tested using the KOBAS program.

### 2.2. DEGs Confirmation Using RT-qPCR

Eight heat-stress-responsive genes were randomly shortlisted from the transcriptome data based on their expression levels and involvement in the KEGG pathways. The expression levels of these genes were further accessed via RT-qPCR to validate the transcriptome data. An Applied Biosystem 7300 Real-Time PCR device was used for the validation, performed in an optical 96-well plate. The cDNA synthesized from 1 μg of RNA was diluted 12 fold, and 4 μL of the diluted cDNA was used as a template to perform the RT-qPCR. Primers were designed (Table S1) from the eight selected sequences to detect their expression levels via RT-qPCR using SYBR Green RT-PCR (Roche, San Diego, CA, USA). The reaction system contained SYBR qPCR Mix, cDNA, forward primer, forward primer and ddH2O with volumes of 10 µL, 4 µL, 1 µL, 1 µL, and 4 µL, respectively, totaling a reaction volume of 20 µL. Mulberry β-actin (with the primers β-actin-F: 5′-AGC AAC TGG GAT GAC ATGGAGA-3′ and β-actin-R: 5′-CGA CCA CTG GCG TAA AGG GA-3′) was used as an internal control gene. All reactions were assayed in three biological replicates. The thermal cycling parameters for RT-qPCR were 95 °C for 60 s, followed by 45 cycles of 95 °C for 10 s, 50 °C for 10 s and 70 °C for 10 s. The relative gene expression levels were estimated via the 2^−ΔΔCt^ method. Three biological replicates were used. The melting curve was analyzed to ensure that the amplification was specific.

## 3. Results

### 3.1. RNA-Seq-Based Transcriptomic Profiles of Mulberry (M. alba)

High-throughput sequencing generated 237,655,186 million pairs of raw reads from four libraries (Table 1). An average of 54,664,526 raw reads was recorded for the control group, and an average of 64,163,067 raw reads accounted for the high-temperature-treated group. Clean reads of 233,081,342 were obtained after quality trimming. A uniform error rate of 0.03 was obtained for each sample read using Trimmomatic software. The GC content and Q30 for each library were above 42% and 92%, respectively. Almost 68% of the clean reads were mapped to the reference genome.

### 3.2. Differential Expressed Genes (DEGs) of Mulberry Responsive to Heat Stress

**Figure 1:**
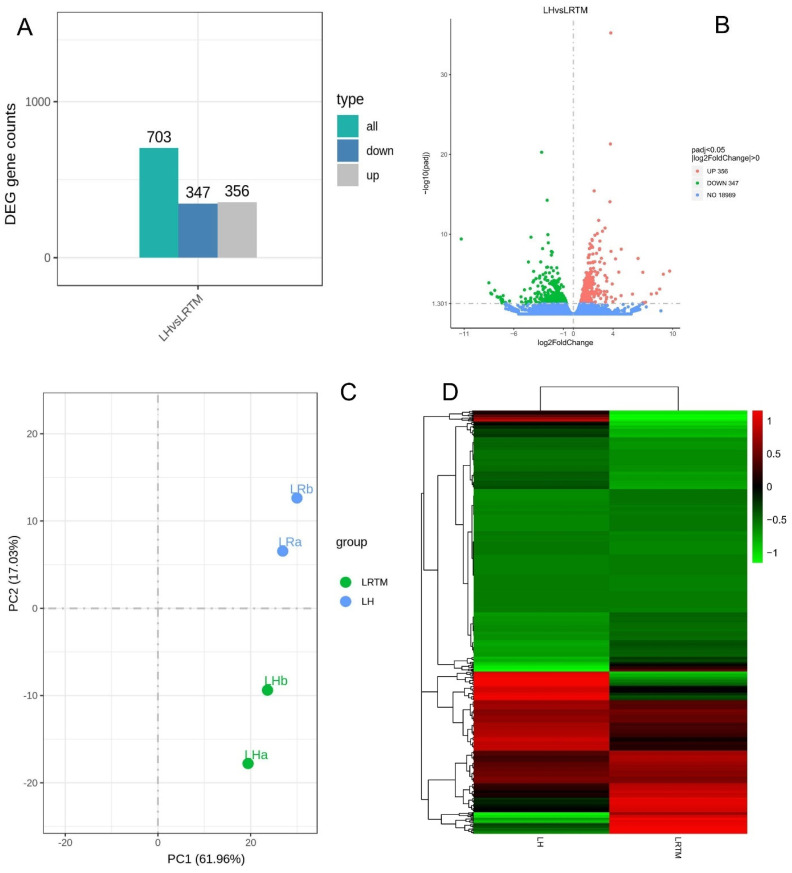
(**A**) Graph depicting the up-regulation and down-regulation of mulberry DEGs in response to heat stress. (**B**) Volcano plot detailing significant gene patterns. Up-regulated genes are shown by red dots, and down-regulated genes are shown by green dots. (**C**) Principal component (PC) analysis. (**D**) Cluster heatmap analysis of the DEGs.

To know the variations in gene expression between the high-temperature-treated group (LH) and the control in response to heat stress, DEGs were analyzed based on the adjusted criteria of *p* < 0.05 and log2 fold change of >0.0 (Figure 1). The up- and down-regulated DEGs in LH vs. LRTM are represented by a bar chart (Figure 1A). The volcano graph illustrates 703 significant differential expression genes. A total of 356 genes were up-regulated, and 347 genes were down-regulated (Figure 1B). Among the genes that were significantly up-regulated were aldehyde dehydrogenase family 3 member F1, protein SIEVE ELEMENT OCCLUSION B, serine/threonine-protein kinase transcript variant X1, chaperone protein dnaJ 11 2C chloroplastic and uncharacterized LOC21395589; these genes had *p*-values of 3.28 × 10^−40^, 5.51 × 10^−26^, 8.29 × 10^−20^, 2.88 × 10^−18^ and 7.21 × 10^−16^, respectively. The down-regulated gene with significant *p*-values included probable aquaporin PIP1-2, BURP domain protein RD22-like, aspartyl protease family protein 22C transcript variant X2 and protein EXORDIUM-like 2. Interestingly, principal component (PC) analysis showed a clear separation of transcriptomes of the two treatment groups, recording a 61.96% variation (Figure 1C), indicating that high temperatures significantly changed the transcriptome of *M. alba*. Moreover, a cluster heatmap was used to reveal the expression pattern of the DEGs in the two groups (Figure 1D).

### 3.3. GO and KEGG Classification of the DEGs

**Figure 2:**
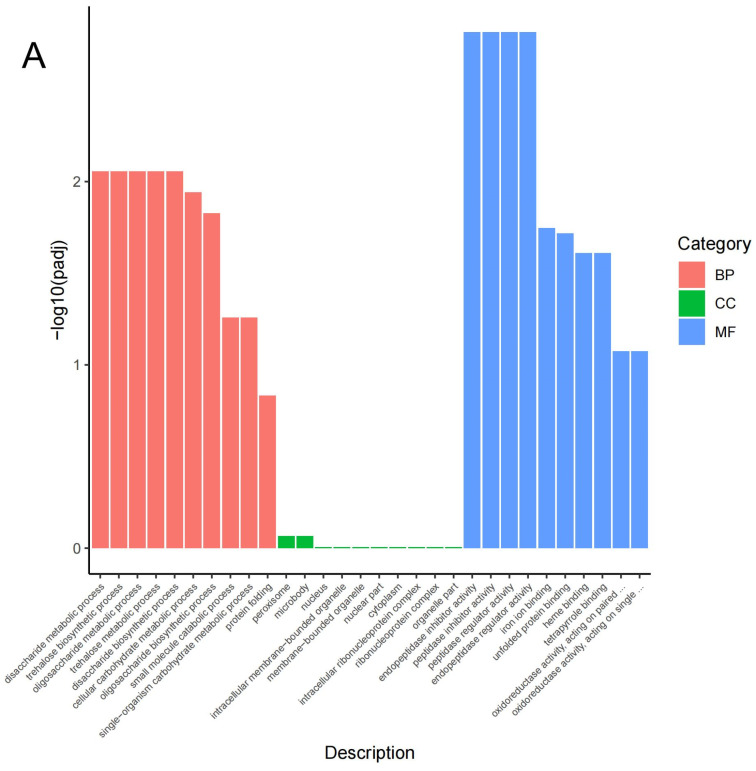

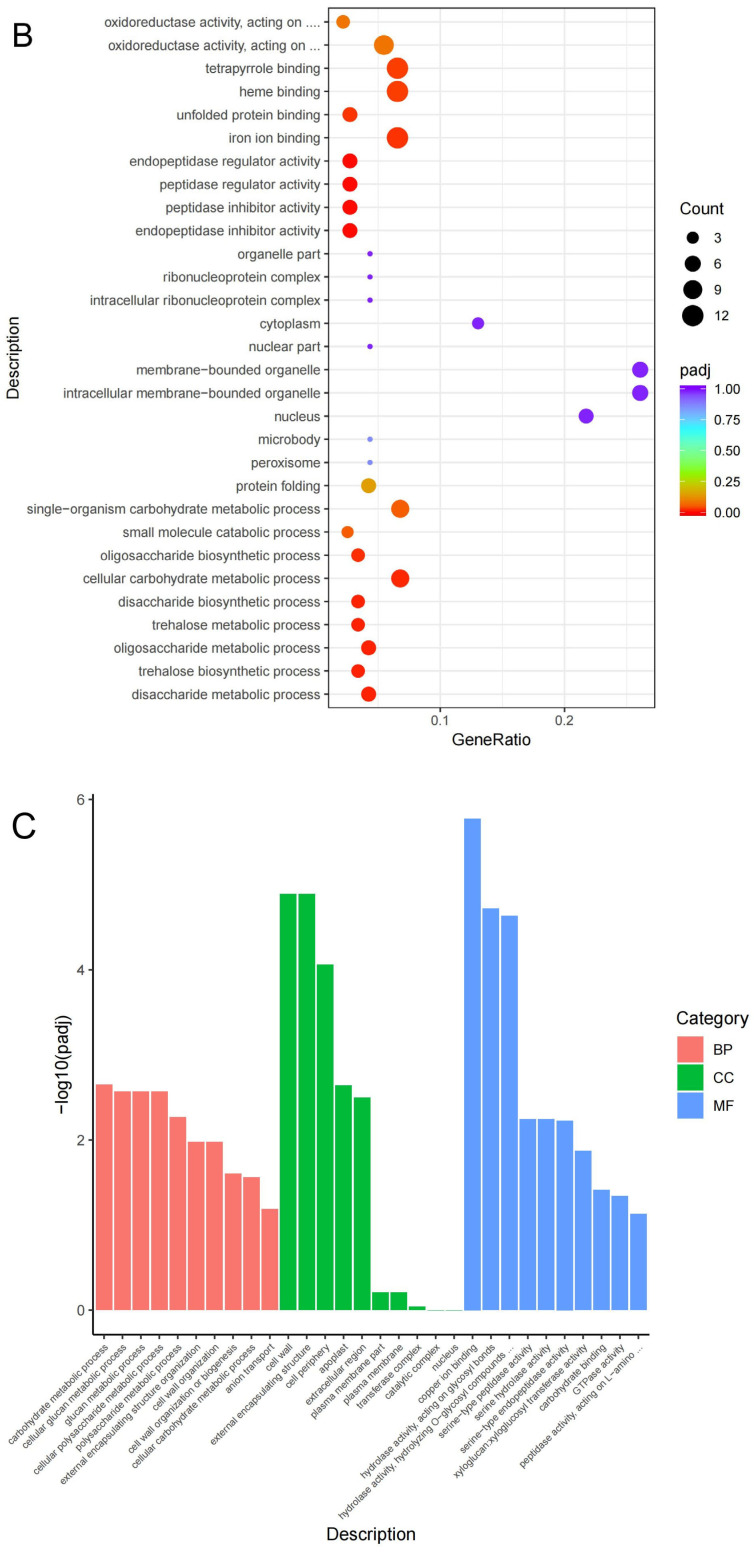

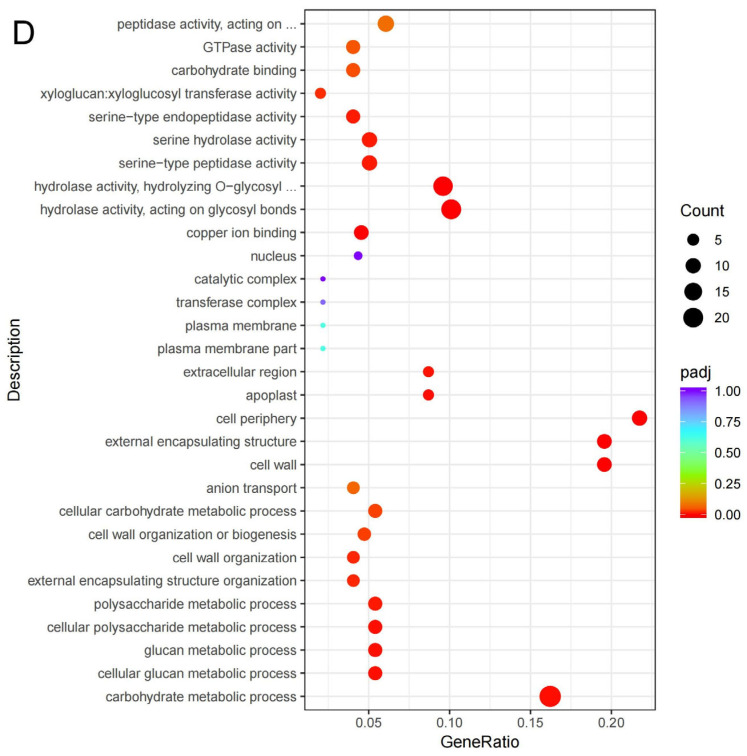
Visualization of GO enrichment terms related to heat response: (**A**) Up-regulated DEGs enriched in GO term classifications. (**B**) GO terms based on enrichment factor in up-regulated genes. (**C**) GO term classification enriched with down-regulated DEGs. (**D**) GO terms based on enrichment factor in down-regulated genes. BP: biological process, CC: cellular component, MF: molecular function.

GO and KEGG enrichment analyses were carried out to further characterize the functions of the DEGs. Based on a *p*-value of *p* < 0.05 and biological significance, 95 of the up-regulated DEGs were assigned to 3 main GO categories and 30 GO terms (Figure 2). These DEGs were more implicated in the biological process category and the molecular function category (Table S2). Among these were the disaccharide metabolic process, cellular carbohydrate metabolic process, tetrapyrrole binding, iron ion binding, etc. (Figure 2A,B). Furthermore, 215 down-regulated DEGs were significantly enriched in 3 GO categories, and also involving 30 GO terms (Figure 2C,D). The GO terms that were enriched with these genes included the cellular carbohydrate metabolic process, carbohydrate metabolic process, cell wall, cell periphery, hydrolase activity acting on glycosyl bonds, hydrolase activity, hydrolyzing O-glycosyl compounds and copper ion binding (Figure 2A). The top 30 enrichments revealed that several of the DEGs were significantly enriched in the GO terms of the cell wall, cell periphery, etc. (Figure 2D).

**Figure 3:**
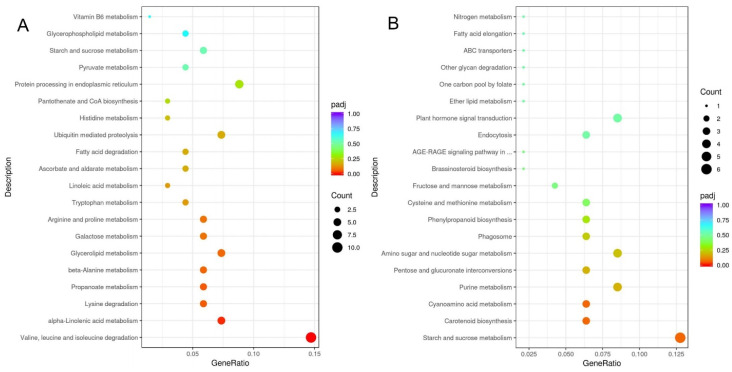
KEGG-enriched DEGs in *Morus alba* at 4 days of heat stress: (**A**) Up-regulated KEGG terms. (**B**) Down-regulated KEGG terms.

Furthermore, analysis of the top 20 of the KEGG enrichment analysis (screening for pathway entries with several differential genes greater than two, sorted by −log10 *p*-value for each entry) showed that DEGs were significantly enriched in valine, leucine and isoleucine degradation (pop00280), and in starch and sucrose metabolism (pop00500), alpha-linolenic acid metabolism (pop00592), carotenoid biosynthesis (pop00906), galactose metabolism (pop00052) and others (Figure 3). Significant down-regulated enriched metabolic pathway included starch and sucrose metabolism (pop00500), carotenoid biosynthesis (pop00906), cyanoamino acid metabolism (pop00460), purine metabolism (pop00230) and pentose and glucuronate interconversions (pop00040).

### 3.4. Heat-Responsive Transcription Factors

Twenty-nine genes that encode ten (10) different TF families were identified in this study. Among these genes, 21 were up-regulated, and 8 genes were down-regulated. The most enriched TF family was DnaJ, with seven genes encoding it. It is significant to note that all seven genes encoding the Dnaj family of TFs were up-regulated. All five (5) genes that encode the AP2 family of TFs were up-regulated. Four of the seven (7) genes that encode MYBs were up-regulated. HLH, WRKY, HSF, Bzip, GATA and TCP TFs were also identified (Table 2).

### 3.5. Validation of DEGs Using Real-Time RT-PCR

**Figure 4:**
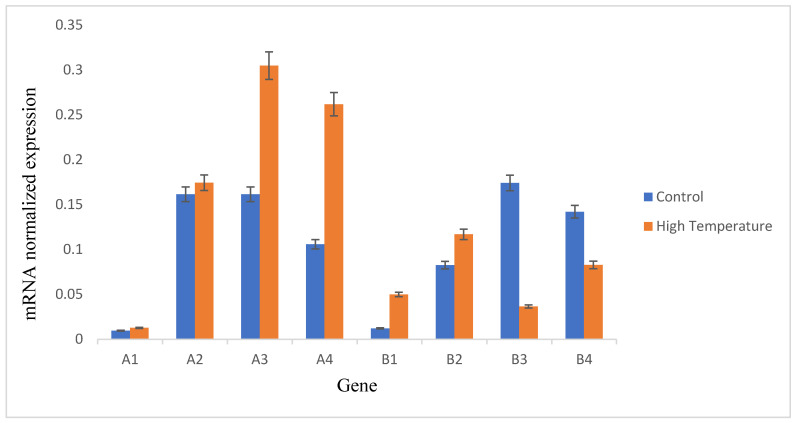
Relative gene expression profile of the eight selected genes in relation to heat stress using RT-qPCR. β-actin served as a reference gene, and the statistical method 2^−(ΔΔCT)^ was applied to obtain the relative quantification. Data represent the average from three biological replicates. A1 = probable polygalacturonase, A2 = chaperone pro dnaJ 11, A3 = heat shock protein 83, A4 = heat shock protein peroxisomal, B1 = cysteine-rich and transmembrane domain-containing protein WIH1, B2 = aldehyde dehydrogenase family 3 members, B3 = subtilisin-like protease SBT4, B4= heavy metal-associated isoprenylated plant protein 7-like.

To authenticate the RNA-Seq data, eight DEGs associated with heat stress (probable polygalacturonase, chaperone pro dnaJ 11, heat shock protein 83, heat shock protein peroxisomal, cysteine-rich and transmembrane domain-containing protein WIH1, heavy metal-associated isoprenylated plant protein 7-like, subtilisin-like protease SBT4 and aldehyde dehydrogenase family 3 member F1) were chosen for analysis with qRT-PCR (Figure 4). The expression of probable polygalacturonase, heat shock protein 83 and cysteine-rich and transmembrane domain-containing protein WIH1 were significantly up-regulated in response to heat, and heavy metal-associated isoprenylated plant protein 7-like was significantly down-regulated in response to heat. The RT-qPCR findings confirmed that the gene expression profiles matched the transcriptomic data.

## 4. Discussion

Temperature stress causes damaging consequences in cell division, leading to a detrimental effect on growth and development [21]. Mulberry seedlings experience drastic morphological changes when exposed to a prolonged period of high temperature, which leads to the shedding of leaves. To increase mulberry’s heat tolerance, it is essential to investigate the genes and transcription factors that respond to heat stress to better comprehend how the plant responds and adapts to heat. However, no findings have been made on the transcriptomic profile of mulberry concerning heat stress. This study examines the effect of heat stress on the mulberry transcriptome using Illumina HiSeq_2000. Furthermore, the FPKM value was calculated to quantify its expression abundance and variations, using RSEM [19]. RSEM (RNA-Seq by Expectation Maximization) is reliable and can be used in analyzing RNA-Seq data even with fewer replicates. The limitation to using RSEM is that, if the reference genome is not available, the user must supply the RSEM in a fasta file for the transcript sequence.

### 4.1. Transcriptomic Changes in M. alba under High Temperature

Some significant DEGs were revealed through the Gene Ontology (GO) analysis. The most significant biological process (BP) term of the gene ontology was involved in the cellular carbohydrate metabolic process. Moreover, 16 genes were involved in the cellular carbohydrate metabolism process. Eight were up-regulated, and eight were down-regulated. Alpha trehalose-phosphate synthase was the most abundant among the eight up-regulated genes, and xyloglucan endotransglucosylase was abundant among the down-regulated genes. Xyloglucan endotransglucosylase has been implicated in cell division and growth expansion. Many researchers have isolated and characterized its role in response to stress. For instance, Van Sandt et al. (2007) isolated XTH enzymes from *Allium cepa* and realized that XTH gene expression is correlated with cell expansion [22]. XTH can also remodel the cell wall of *Arabidopsis* in cell division and promote growth in response to stress [23]. Lurlaro et al. (2016) discovered that all XTH expression in thermosensitive wheat is down-regulated, which is consistent with our findings [24]. A contradictory transcriptome report showed that genes encoding proteins from the XTH family in *Brassica rapa*, L. are up-regulated in response to heat stress [25]. According to these results, plant responses to stress are perhaps dependent on the species, plant age, organ and timing and intensity of the stress. In a more recent report, the exposure of wheat to high temperatures for 15 days up-regulated the relative expression levels of XTH1, XTH2 and XTH5 using RT-qPCR analysis [26]

Trehalose (α-glucopyranosyl-α-d-glucopyranoside) is found in most organisms, including plants, bacteria, fungi and invertebrates. It comprises two glucose molecules and is produced rapidly in response to various cellular stresses. Trehalose is implicated in protecting yeast cells by increasing their resistance to internal and external stresses. The disaccharide appears to increase resistance to heat [27]. Trehalose is also known to prevent or inhibit denatured proteins from aggregation, allowing the reactivation of molecular chaperones such as HSFs. The up-regulation of genes encoding this enzyme could suggests that, in the presence of heat stress, *M. alba* responds by elevating the enzyme to prevent the aggregation of denatured proteins caused by the heat to activate other heat shock proteins. Several studies have reported the overexpression of trehalose-activated genes in response to abiotic stress, leading to an increase in trehalose and proline content [28,29].

The up-regulated genes involved in the hydrolase activity under the molecular function (MF) category of the gene ontology are alkaline/neutral invertase (A/N-INV), beta-D-xylosidase and polygalacturonase. Developing literature has highlighted the prospective role of A/N-INVs in plant development and its response to environmental stimuli and abiotic stress in various plant species, such as *Arabidopsis thaliana* [30] and *Oryza sativa* [31]. For example, the regulation of cellular hexose concentration by *A. thaliana* AtCYT-INV1 is essential for plant growth and osmotic stress inhibition. The PtrA/NINV gene from the trifoliate orange is a stress-responsive gene because its expression is triggered by multiple stresses, such as cold and salt stress [32]. A/N-INV up-regulation in our findings suggests that it may play a vital role in stress tolerance under heat stress. A study on tobacco under abiotic stress (cold) revealed that genes involved in A/N-INV metabolism are significantly up-regulated [33], indicating the significant role of gene expression in this study.

Polygalacturonase belongs to one of the most prominent hydrolase families. Polygalacturonase (PG) is a crucial enzyme that participates in numerous plant growth and developmental processes, including flower development, fruit ripening and senescence [34,35]. Polygalacturonase (PG) is implicated in pectin degradation during fruit ripening, organ aging and plant stress responses [36]. Upon the treatment of rice plants with cold, salt and drought conditions, OsBURP16 (family ofPG1β) is up-regulated [36]. Furthermore, an optimum temperature of 40 °C or higher for polygalacturonase stability has been reported [37]. Our results, together with the previous reports about polygalacturonase activity concerning high temperatures, suggest that it plays a role in heat stress tolerance. Future research will be conducted to confirm the enzyme’s activity concerning heat stress in *M. alba*.

### 4.2. Heat Stress Response in Mulberry Induced Up-Regulated Genes Related to Valine, Leucine and Isoleucine Degradation

**Figure 5:**
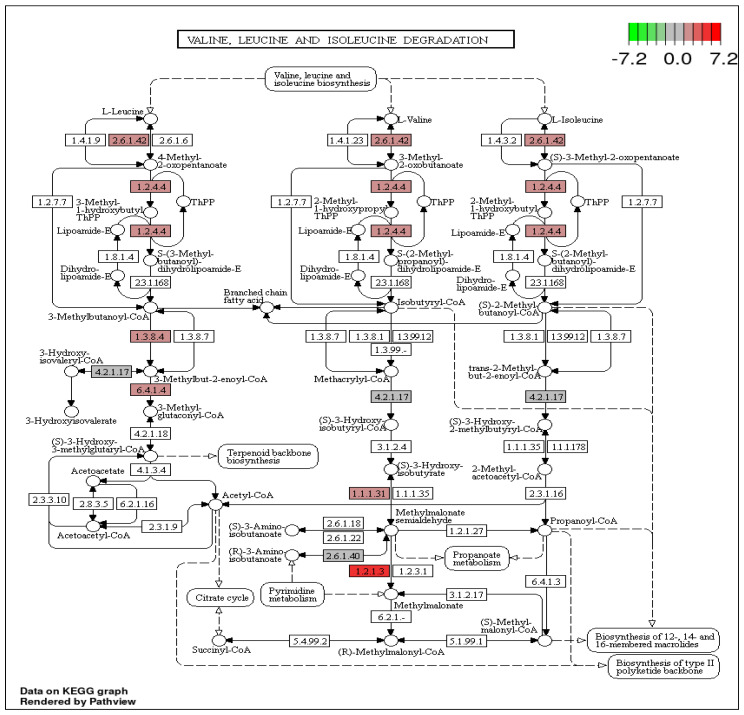
KEGG map showing DEGs in the valine, leucine and isoleucine degradation pathways of *M. alba* under heat stress. A red color represents up-regulated genes, and deeper red colors indicate the up-regulation of expression levels. The codes in the rectangular boxes represent the IDs of the enzymes that the genes encode.

Our study revealed that 12 genes participated in the valine, leucine and isoleucine degradation pathway. They included alanine--glyoxylate aminotransferase (LOC21404820), 2-oxoisovalerate dehydrogenase subunit alpha (LOC21403577), methylcrotonoyl-CoA carboxylase beta chain (LOC21410135), 2-oxoisovalerate dehydrogenase subunit beta 1 (LOC21388555), aldehyde dehydrogenase family 3 (LOC21398387), probable 3-hydroxybutyrate dehydrogenase (LOC21408668), branched-chain amino acid aminotransferase 1 (LOC21390509), isovaleryl-CoA dehydrogenase (LOC21397545), methylcrotonoyl-CoA carboxylase subunit alpha (LOC21385626), probable enoyl-CoA hydratase 1 (LOC21392597), aldehyde dehydrogenase family 3 (LOC21396538) and alanine--glyoxylate aminotransferase (LOC21404820). In plants, the catabolism of amino acids is extremely important in metabolic stress (e.g., when there are limited carbohydrates during prolonged darkness or any other stress). In these circumstances, amino acids are often used as substitutes for respiration. Total oxidation in the mitochondria of BCAA leucine, isoleucine (Ile) and valine (Val) effectively facilitates the formation of ATP by oxidative phosphorylation. The catabolism of amino acids is particularly important in terms of germination (conversion of storage proteins into carbohydrates), senescence (recycling of energy-rich compounds) and stress reactions [38]. Drought and salt stress significantly cause the synthesis of specific amino acids [39]. Those amino acids are rapidly depleted upon the release of stress. Aminotransferases are involved in multiple metabolic pathways, including the metabolism of amino acids, assimilation of nitrogen, gluconeogenesis, responses to a variety of biotic/abiotic stresses and other pathways [40,41]. ß-Alanine is a known plant osmoprotectant. Putative 3-hydroxybutyrate dehydrogenase, AtHDH1 (At4g20930), is involved in Val and Ile degradation [42]. In our study, most genes that encode enzymes that are involved in the valine, leucine and isoleucine degradation pathways were up-regulated in response to heat stress (Figure 5). This indicates that the valine, leucine and isoleucine degradation pathways play a significant role in the heat stress response in mulberry (*M. alba).* Under heat stress treatment, amino acids such as proline are up-regulated, whereas lysine, tyrosine and aspartic acid are down-regulated in the amino acid metabolism, when *Apium graveolens* is exposed to heat stress [43].

### 4.3. TFs Responsive to High Temperature

Transcription factors play a role in many biological processes, including hormone signaling, organ formation, metabolism and responses to biotic and abiotic stresses. In the presence of external stresses, TFs cause a relay of signaling in plants that directly or indirectly regulate target genes to respond to the stress. Our findings reveal that a sizable number of transcriptional factors responded to heat stress, confirming that TFs are indeed crucial for triggering stress-responsive genes to either adapt to or reduce the effect of the stress. Notably, many researchers have adequately explained the significance of TFs involved in heat stress to activate or repress the transcription of heat stress (HS)-inducible genes [44]. TFs such as the DnaJ, NAC, HSF, MYB, AP2, GATA, WRKY, bHLH and TCP families were primarily identified as mulberry responded to high temperatures.

DnaJ proteins are ubiquitous in all plant species and are essential molecular chaperones. They are also involved in signal transduction and cellular protein homeostasis and are tolerant to abiotic stresses. AtDjA2 and AtDjA3, two DnaJ protein gene homologs, have improved heat resistance in *Arabidopsis* [45]. The chaperone protein DNAJ11 is a member of the heat shock protein (Hsp 40) family. Under stress conditions, plants use the majority of Hsps in the peroxisomal matrix to avoid the accumulation of partly denatured proteins [46]. Heat shock proteins protect against biotic and abiotic stresses. Besides this, Hsps increase membrane stability and neutralize reactive oxygen species by alternating the activities of antioxidant enzymes [47]. HSFs regulate plant resistance to anoxia, heat, osmotic and oxidative stresses [48]. In *Arabidopsis* plants, the expression of a thermosensitive male sterile (TMS1) DnaJ protein confers thermotolerance to pollen tubes [49]. Significantly, the expression of AtDjB1 improves thermotolerance in *Arabidopsis* by shielding cells against heat-induced stress injury [50]. In our study, the expression profile of the DnaJ family suggests that the 42 °C heat stress induced downstream genes to curb the external heat stress. A more recent article revealed that the transcriptome profile of *Aegilops speltoides* under heat stress induces higher expression levels of TFs, including several Hsps [51]. Sohrabi and colleagues revealed that several TFs, including AP2/ERF and bHLH, are up-regulated when lentils are exposed to heat stress [5].

AP2/ERFs react to multiple hormones and environmental stresses [52]. AP2/ERF significantly impacts plant growth and responds well to cold, heat, salt and droughts [53]. AP2/ERF reacts to heat-responsive genes; for instance, the overexpression of the ERTI gene from *Arabidopsis* improves its tolerance to heat, drought and salt stresses [54]. All the genes that encode AP2 in this study were up-regulated [5], suggesting that heat stress induces AP2 transcription factors to activate downstream genes to respond to the stress.

Data from diverse plant species show that MYB family members partake in plant responses to temperature stresses (cold and heat). A transcriptome study of MYB genes and their expression in *Arabidopsis and Oryza sativa* suggested the tentative role of most MYB domain proteins in response to abiotic stress [55]. Most of our study’s MYB genes were up-regulated; however, a significant number of its members were also down-regulated, which agrees with a study reporting on *A. speltoides*, which reported that several TFs, including HSF, WRKY, MYB, AP2, bZIP and bHLH, exhibi both up- and down-regulation patterns under heat stress [43]. The reason for the down and up-regulation needs to be further investigated. NAC is a large family of transcription factors involved in plant developmental processes and responds well to abiotic stresses. ABA is mostly induced by NAC29 and NAC72, which subsequently respond and mitigate stress [56]. NAC 72 targets (PUB19, ATHB13 and BAM1) are noted to participate in diverse abiotic stress responses in *Arabidopsis* [57]. Therefore, NAC72 identified in this study could be a significant regulator of ABA signaling to control heat stress in mulberry.

WRKYs have various plant activities; they promote plant growth and development, synthesize secondary metabolites and respond to biotic and abiotic stress by regulating numerous genes [58]. The constitutive overexpression of AtWRKY25 and AtWRKY26 improves heat stress resistance in *Arabidopsis* [59]. Apart from the above discussed TFs, GATA, HSF, transcription factor 8, MADS-box transcription factor 23, Zn Finger, BHLH and TCFs were also discovered, implying that many TFs are activated as transcriptional regulators to trigger downstream target genes in response to high stress.

Under stress conditions, plants use the majority of Hsps in the peroxisomal matrix to avoid the accumulation of partly denatured proteins [46]. Aldehyde dehydrogenases (ALDH) are a group of enzymes involved in plant metabolism and help in aldehyde homeostasis to remove toxic aldehydes. ALDH enzymes, in their enzymatic reactions, generate NADPH and NADH and thus help in the balancing of redox equivalents. They are involved in stress adaptations to biotic and abiotic environments and regulate aldehyde homeostasis under stress conditions. For instance, ALDH3I1 and ALDH7B4 from *Arabidopsis* are strongly induced by heat stress [60].

Validated DEGs down-regulated in RT-qPCR analysis include heavy metal-associated isoprenylated plant protein 7-like and subtilisin-like protease SBT4. HIPPs (heavy metal-associated isoprenylated plant proteins) are metallochaperones that contain a metal-binding domain (HMA) and C–terminal isoprenylation [61]. Most HIPPs have one or two HMA domains and an isoprenylation motif and regulate genes to respond to cold and droughts [62]. Subtilisin-like proteases (subtilases) are serine proteases in plants that perform a specific function in plant development and signaling cascades [63]. Proteolysis is important for the normal functioning of multicellular organisms and performs critical functions in a number of processes, such as growth, physiology, defense responses, responses to stress and adaptations to changing environments [63].

### 4.4. Validation of the RNA-Seq Results by qRT-PCR

To determine the reliability of the DEGs obtained in this study, eight DEGs linked to abiotic stress were chosen, and their expression levels were evaluated using RT-qPCR. The findings of RT-qPCR revealed that the expression patterns of the majority of these DEGs confirm the data obtained from the Illumina sequencing analysis. (Figure 4). However, the results for cysteine-rich and transmembrane domain-containing protein WIH1 did not match those from RNA-Seq. The up-regulated DEGs that twere validated through RT-qPCR analysis were polygalacturonase, chaperone pro dnaJ 11, heat shock protein 83, heat shock protein peroxisomal, aldehyde dehydrogenases and cysteine-rich and transmembrane domain-containing protein WIH1.

The findings of RT-qPCR revealed that the expression of most of these DEGs is similar to that obtained from the Illumina sequencing analysis. The transcriptome result is a true profile of heat-stressed mulberry.

## 5. Conclusions

We discovered an extensive transcriptome profile of heat-stressed mulberry using RNA-Seq technology (Novogene, Beijing, China). Overall, 703 DEGs (356 up-regulated and 347 down-regulated) were found under this standard (corrected *p*-value < 0.05, log2fold change >0.0) in mulberry. Several genes were identified related to valine, leucine and isoleucine degradation, and to starch and sucrose metabolism, alpha-linolenic acid metabolism and plant hormone signal transduction under high temperatures. In addition, TFs such as AP2/ERF, WRKY, HSF, bHLH, NAC, MYB, TCP and others were also implicated in abiotic (heat) stresses. We suggest that a functional study should be conducted on mulberry xyloglucan endotransglucosylase/hydrolase, alpha, alpha trehalose-phosphate synthase, alkaline/neutral invertase D, beta-D-xylosidase and polygalacturonase to ascertain their functional activities with respect to heat stress. Moreover, the valine, leucine and isoleucine degradation pathways play a vital role in mulberry heat responses. Future research should focus on these pathways to reveal their tentative roles. The results of this analysis provide a theoretical basis and can be used to make valuable comparisons in future research on the heat tolerance of mulberry plants and other plant species.

## Figures and Tables

**Table 1:** Summary of quality preprocessing of RNA-Sequencing data.

Sample ID	Raw Reads	Clean Reads	Clean Bases	Error Rate	Q30	GC%
LRa	57,243,672	56,159,618	8.4 G	0.3	92.67	44.29
LRb	52,085,380	51,122,510	7.67 G	0.3	92.5	44.63
LHa	66,765,656	65,546,612	9.83 G	0.3	92.89	44.65
LHb	61,560,478	60,252,602	9.04 G	0.3	92.84	44.43

LRa + LRb = Control (LRTM). LHa+ LHb = High-temperature group (LH).

**Table 2:** Heat-responsive transcription factors identified during the analysis.

TF Family	Gene ID	Up/Down
Dnaj	21394896, 21401054, 21395794, 21393813, 21398174, 21405132, 112091504	Up
AP2	21407265, 21406253, 21398364, 21392224, 21387978	Up
MYB	21406818, 112092520, 21399045, 21394542 21394127, 21398688, 112093121	Up Down
HLH	21404443, 21404618, 21402833	Up Down
NAC	21389501 21397328	Up Down
Bzip	112092685	Up
WRKY	21404214	Up

## Data Availability

The original datasets described in this study are included in the article/Supplementary Materials. The raw seq data used for this article can be found in the NCBI SRA database with accession number PRJNA953831. Further inquiries can be addressed to the corresponding author.

## Supplementary Materials

The following supporting information can be downloaded at https://www.mdpi.com/article/10.3390/cimb45050264/s1: Table S1: Designed primers for the qRT-PCR; Table S2: GO enrichment.
