# Supplementary material for: Genome-Wide Identification of Candidate Genes Associated with Heat Stress in Mulberry (Morus alba L.)
Source: Curr Issues Mol Biol. 2023 May 8;45(5):4151–67. doi: 10.3390/cimb45050264 (PMC10217537; doi:10.3390/cimb45050264)
Supplement: Supplementary file 1 [file cimb-45-00264-s001.zip › Table S1.pdf]

Table S1. Designed primers for the qRT-PCR

Gene name	Gene description	Primers
LOC21404334	Probable Polygalacturonase	F-CCTGTGCTCAAACCTTTGCC<br>R-TTTCCCCGGTAACGACGATG
LOC21407693	chaperone pro dnaJ 11	F-GCGAGAACTTGTCACCCTGA<br>R-CAGGATCGGACAACGTGGAA
LOC21406971	heat shock protein 83	F-GGCAACTCGTCAACAAGCAG<br>R-TTCCCAGTCATTGGTGAGGC
LOC21388509	heat shock protein, peroxisomal	F-AGGTGAGAAAGAGGAGTCCCA<br>R-CCGCCTTTACATTTTCCGGC
LOC21392711	Cysteine-rich and transmembrane domain-containing protein WIH1	F-AAGGACGCCTACCCTCAAAC<br>R-CAAAGTGCCGCCAAGCATC
LOC112094770	Heavy metal-associated isoprenylated plant protein 7-like	FCAAAGGTGACAGTGAAGGGAGA<br>R-GGAGGTTTTGGGATTGGAGAG
LOC21403666	Subtilisin-like protease SBT4.14	F-TGACTACACGGCGGACTCTA<br>R-AGCCACAGTCAGTAACCACG
LOC21398387	Aldehyde dehydrogenase family 3 member F1	F-GTCGGCGAACAACTCCTACA<br>R-CACCAAGCTCCAAAGCAACC
